# A Prevalent Affection Much Overlooked

**Published:** 1888-06

**Authors:** E. Meierhof

**Affiliations:** New York


### A PREVALENT AFFECTION VERY MUCH OVERLOOKED.

*By E. Meierhof, M. D., New York.*

For Daniel's Texas Medical Journal.

The frequency with which the writer meets adenoid vegetations in the superior vault of the pharynx, especially in children, is the occasion for these lines. Every practitioner meets cases of this kind. Children of the age from three to twelve years, complain of coughing, the cough is generally of a dry, hacking kind, or may be mucous in character, respiration during sleep is entirely by the mouth, accompanied with more or less loud snoring, the voice is thick, or, as frequently erroneously termed nasal in tone. In moving about the mouth is usually open. The above clinical picture will be given by the parents of such children.

If the nasal chambers can be inspected it will be found that nasal respiration is often obstructed. The nose is filled with more or less mucous. If the patient is tractable turgescence of the mucous membrane lining the nasal chambers will be seen, occasionally a hyperæmia alone is present, etc. The examination thus far deals with the anterior nares. Inspection of the throat frequently reveals nothing unusual. Enlarged tonsils are at times met with, and often not, the pillars of the fauces may or may not be reddened. On the posterior wall of the pharynx mucous is frequently seen. I'll leave the examination of the posterior nares and superior vault of the pharynx for a later consideration. An examination of the chest gives only negative signs except we may find an occasional bronchitis. In a large number of cases frictional breathing is frequently heard, otherwise the patient in this respect may appear healthy, appetite is capricious, the spirits of the child is also interfered with, and at times there is positive mental hebetude, the stature instead of being erect or nearly so becoming bent if the trouble is of long continuance. Temperature and pulse remaining normal. The patient has been dosed with cough and throat mixtures, the nose syringed or sprayed, counter-irritation applied to the various regions of the neck; sometimes a vermifuge is given to drive out a suspected worm in the stomach or throat, and yet the patient goes on as before, "left to grow out of his trouble," coughing at night and snoring loud enough to awaken himself; becoming frightened at his own disturbance. Such cases as these I frequently meet, and I believe, so do you. The cause for the production of this trouble has been overlooked, owing to the fact that it is hidden from ordinary view. Anyone who makes examinations of the posterior nares with a mirror knows the difficulties which frequently beset him, owing to anatomical and other causes; this is with adults, how much more difficult is it with children! To examine the rhino-pharynx in children there is but one method practicable in all cases and that is the use of the index finger. The touch sense becomes as acute and intelligent as is necessary in making gynæcological examinations. The patient may resist but this need not deter you in the least, as the jaws may be kept apart by a regular mouth-gag, the use of a twisted portion of a towel introduced between the jaws, or by standing up with the patient's face against your body, using either the right or left hand as convenience may dictate, force the cheek between the jaws and the patient cannot close down on you whilst the examining finger of the other hand is pushed into the mouth, feeling for the uvula, and then pass the finger behind this structure ; feeling above on the superior pharyngeal wall, sweeping the finger laterally to one side and then to the other, and lastly feel in the posterior nares, etc. By doing this, hypertrophies of the turbinated structures, adenoid vegetations, etc., can be made out. Regarding the turbinated hypertrophies, I leave these to confine myself to the consideration of adenoid vegetations, or hypertrophy of the pharyngeal tonsil of Luscha, which is situated in the superior vault of the pharynx. These morbid growths according to a number of observers, including the writer, are the cause for the clinical picture given above. It is to Meyer, of Copenhagen, that we are indebted for the progress made in investigating this cause of many of the disturbances produced by adenoid vegetation. When the finger is introduced behind the soft palate to examine the condition of the mucous membrane lining the naso-pharynx, the presence of these vegetations may be recognized by the soft and yielding nature of the structures to the touch. In some cases the mass is quite abundant and in others less so, however, the amount of the growth is not always an indication of the extent of disturbance produced.

In some cases where the mass was quite large the disturbance was not so great as in others when it existed in a lesser degree. It is the writer's sincere conviction that the extent of the prevalence of adenoid vegetations must be great. The causes for the production of the growths are evidently due to numerously repeated congestions of the naso-pharynx which finally render the mucous membrane, and its blood vessels lax, thereby interfering with the normal blood circulation finally producing varicose structures such as occur elsewhere when the circulation is obstructed passively, thereby producing an unusual richness of vascular supply which finally gives rise to these vegetations, and they in turn secrete mucus mixed with the epithelial cells, which becoming thickened, interfere with respiration, producing trophic and other disturbances. The importance of this subject can be well judged from the fact that a meeting of the laryngological section of the New York Academy of Medicine, on Februaay 21, was devoted entirely to this subject. There were present a large number of gentlemen; Boston, Brooklyn, Baltimore, besides New York, were represented. Some of the specimens handed around by Dr. Hooper, of Boston, astonished even those that have given the subject much attention, and it seemed as if Bostonian children were afflicted with this trouble to a greater degree than those met in New York. Dr. C. Blake, of Boston, spoke of their connection in the production of aural disease, etc.

While this article is a very incomplete one, and does not do full justice to the subject, I wish to add a few words regarding its treatment. Various instruments have been devised for the removal of these growths, such as curetts, sharp spoons, spoon-jawed forceps, gulvano cautery points of special device, guillotines, etc., which are more satisfactorily used on older children, but which are more or less unsatisfactory in young children, except as Dr. Hooper does, anœsthetises his patient for the operation, using the spoon-jawed forceps of Lowenberg to remove the mass. This requires some skill and experience, and is not entirely free from danger in the production of acute purulent otitis, therefore I wish to call attention to the use of the examining finger as a means in the treatment or removal of these masses by merely crushing. It seems unnecessary to state that the finger should be immaculate in cleanliness, with the nail pared. If necessary a twisted towel can be used as a gag. I rarely used anything, except to press the cheek between the jaws if there is any resistance. The right or left finger is introduced by the soft palate, the patient sitting or standing, according to the convenience of the operator. My patients usually are seated. Feel rapidly for the vegetations, at the same time pressing the finger into the masses if any is felt, with sufficient force to crush them; they cannot all be crushed at one sitting, but this procedure can be repeated every four or five days, until the soft gelatinous masses cannot be felt; the finger must be swept all over the naso-pharynx laterally as well as superiorily in search of these structures, at the same time crushing. Immediately after an operation, profuse bleeding will take place through the nose and mouth which need not cause any surprise or alarm. This bleeding in a lesser degree will take place at subsequent operations until at last there is little or no hæmmorrhage. Frequently after the first operation by this method alone improvement takes place, and generally continues until the symptoms more or less disappear.

